# Wildlife fecal microbiota exhibit community stability across a longitudinal semi-controlled non-invasive sampling experiment

**DOI:** 10.3389/frmbi.2024.1274277

**Published:** 2024-02-13

**Authors:** Samuel B. Pannoni, William E. Holben

**Affiliations:** ^1^ Franke College of Forestry and Conservation, University of Montana, Missoula, MT, United States; ^2^ Cellular, Molecular and Microbial Biology Program, University of Montana, Missoula, MT, United States

**Keywords:** microbiome-stability, non-invasive, wildlife, longitudinal time-series, 16s-rDNA sequencing, LiMM-PCA

## Abstract

Wildlife microbiome studies are being used to assess microbial links with animal health and habitat. The gold standard of sampling microbiomes directly from captured animals is ideal for limiting potential abiotic influences on microbiome composition, yet fails to leverage the many benefits of non-invasive sampling. Application of microbiome-based monitoring for rare, endangered, or elusive species creates a need to non-invasively collect scat samples shed into the environment. Since controlling sample age is not always possible, the potential influence of time-associated abiotic factors was assessed. To accomplish this, we analyzed partial 16S rRNA genes of fecal metagenomic DNA sampled non-invasively from Rocky Mountain elk (*Cervus canadensis*) near Yellowstone National Park. We sampled pellet piles from four different elk, then aged them in a natural forest plot for 1, 3, 7, and 14 days, with triplicate samples at each time point (i.e., a blocked, repeat measures (longitudinal) study design). We compared fecal microbiota of each elk through time with point estimates of diversity, bootstrapped hierarchical clustering of samples, and a version of ANOVA–simultaneous components analysis (ASCA) with PCA (LiMM-PCA) to assess the variance contributions of time, individual and sample replication. Our results showed community stability through days 0, 1, 3 and 7, with a modest but detectable change in abundance in only 2 genera (*Bacteroides* and *Sporobacter*) at day 14. The total variance explained by time in our LiMM-PCA model across the entire 2-week period was not statistically significant (p>0.195) and the overall effect size was small (<10% variance) compared to the variance explained by the individual animal (p<0.0005; 21% var.). We conclude that non-invasive sampling of elk scat collected within one week during winter/early spring provides a reliable approach to characterize fecal microbiota composition in a 16S rDNA survey and that sampled individuals can be directly compared across unknown time points with minimal bias. Further, point estimates of microbiota diversity were not mechanistically affected by sample age. Our assessment of samples using bootstrap hierarchical clustering produced clustering by animal (branches) but not by sample age (nodes). These results support greater use of non-invasive microbiome sampling to assess ecological patterns in animal systems.

## 1 Introduction

The advent of high-throughput DNA sequencing capabilities has led to a growing number of studies relating the composition of microbiomes to the condition of their hosts and the host’s environment (Wu et al., 2011; Taschuk and Griebel, 2012; Hale et al., 2016; Gaulke et al., 2018; Knutie et al., 2019; Lynch and Hsiao, 2019; Corl et al., 2020). The gold standard of capture-based (i.e. direct) fecal sampling of a host animal is ideal for limiting the influence of abiotic factors on microbiome composition (Carroll et al., 2012). However, the growing need for studying rare, endangered, or elusive species dictates the use of non-invasive fecal sampling, often many days after defecation (Zhu et al., 2021). Since the ability to control for sample age is not always possible, the potential influence of time-associated abiotic factors should be assessed before interpreting microbiome sequencing results. This type of experimental groundwork is necessary to temper conclusions or inform potential confounding variation when strict sampling regimes are not possible, as is often the case in wildlife microbiome studies.

Gut and fecal microbiomes have a strong coevolutionary bond to their host (Gaulke et al., 2018; Amato et al., 2019). This interaction is especially apparent in ungulates where microbially mediated fermentation occurs in the rumen to produce short-chain fatty-acids (SCFAs), the animal’s primary source of energy (Wang et al., 2020). As such, rumen microbiota composition often reflects specific health attributes of the host, which cascade through the gastrointestinal tract resulting in equally informative, although distinct, fecal microbiota (Ingala et al., 2018; Pannoni et al., 2022).

In comparison to the number of host-microbiome studies available, only a limited number of foundational experiments have been conducted to quantify the influence of potentially confounding environmental factors acting on the microbiome communities prior to and post sampling (Roesch et al., 2009; Tzeneva et al., 2009; Lauber et al., 2010; Cardona et al., 2012; Carroll et al., 2012; Hale et al., 2016; Kim et al., 2017). These studies varied in sample type, study species, length of experimental treatment (hours, days, or weeks), environment, and analysis methods and perhaps not surprisingly presented often-conflicting conclusions. Despite their lack of congruence, these studies and their findings present a useful starting point for the wildlife microbiome practitioner when designing a non-invasive sampling protocol using fecal microbiota to gain insights into host health and ecology. Considering the potential breadth of influential environmental factors acting on non-invasive fecal samples and the apparent lack of consistency in effects between studies, we suggest experimental evaluation of field effects catered to specific study conditions.

Following defecation, fecal microbial communities are exposed to drastic changes in environmental conditions known to alter the growth and survival of anaerobic bacteria, including changes in UV exposure (McCambridge and McMeekin, 1981; Arrieta et al., 2000), temperature (Huus and Ley, 2021), and oxygen (Brusa et al., 1989). It is highly likely that the metabolic activities of these communities shift dramatically due to these changes being more or less favorable to each taxon (Menke et al., 2015). What is less clear is how rapidly the composition within these fecal pellets changes, since changes in abundance require growth and turnover (loss) of taxa in this new environment. Further, the extent to which these various abiotic and biotic influences may be buffered within fecal pellets that have a protective exterior (e.g. formation of a hard, dry exterior “shell” through desiccation), or during winter when low temperatures inhibit microbial metabolism, growth and death, are still open questions. Thus, we hypothesized that fecal pellets dropped during winter or early spring in temperate climes would exhibit stable fecal microbiota composition (as determined by 16S rRNA survey techniques) for some period of time. Herein, we address that hypothesis to better guide the use of non-invasive methods in wildlife studies.

We designed an experiment to quantify the potential bias of sample age (i.e. time since defecation) on fecal microbiota of a North American ungulate, where sampling takes place in late winter/early spring conditions, typically within a 2-4 day window. The purpose was to assess potential community compositional changes through time that might confound experimental observations and interpretation when samples come from an unknown timeline. We sampled fecal pellets from four Rocky Mountain elk (*Cervus canadensis*) non-invasively, *but at the time of defecation*, near Yellowstone National Park, Montana in March 2016. The longitudinal component of this fecal microbiota experiment was conducted in a forested plot near Evaro, MT beginning on the day of collection after transporting the fecal pellet samples in sterile whirl-pak bags on ice. In our design, sample age is controlled by repeatedly subsampling elk fecal pellets originally sampled from the same individual across day 0, 1, 3, 7, and 14, in triplicate. Thus, our hierarchical block experimental design captures compositional fecal microbiota change through time within four elk biological replicates (blocks) and estimates the variation within each block using three technical replicates for each sample/time combination. Hierarchical block design is employed by life science experiments to differentiate between the mean effect of a drug or other factor (i.e., *fixed* effect) without the confounding variation of measuring this in different patients or subjects (i.e., *random* effects) (Gelman, 2005). This design often employs the use of linear mixed models (LMM) to partition the specific error structure associated with non-independence of samples (multiple measures on the same individual across time points) and to control for the high variance within biological replicates (pseudo-replication) that often leads to type-1 error when ignored (Mata et al., 2019).

In this experiment we ask: (i) Are fecal microbiota communities in elk stable across intervals typically used for non-invasive sampling? (ii) Does sample age influence the results of commonly measured diversity estimates and sample clustering analyses of these communities? (iii) What proportion of the variance in microbial composition is attributed to time, individuals, and pseudo-replicates? Quantifying and isolating sources of variation in elk microbiota due to sample age and classification of the microbial taxa driving changes in these ‘semi-controlled’ experiments will allow more thoughtful interpretation of various diversity estimates and differentially abundant taxa in other studies where this potential source of variance (time since defecation) cannot be controlled. We also highlight a longitudinal modeling technique called LiMM-PCA (Linear Mixed Models-Principle Components Analysis) first implimented by Manon and Govaerts, 2020, that is of particular value for microbiome studies which often have hierarchical sampling designs, repeat measures and multivariate responses (i.e., microbial compositions).

## 2 Methods

### 2.1 Collection of fecal samples and experimental setup

Scat samples from 4 elk were collected near the northern boundary of Yellowstone National Park in Montana in March 2016. Animal sampling was conducted non-invasively within 15 minutes of defecation. Elk sex and age could not be accurately determined due to these samples being collected after observing the elk defecating from a distance using binoculars. Based on our observations, they were most likely adult females or young males. Fecal samples from each scat pile (i.e., individual) were collected from the ground with sterile gloves and forceps and placed in sterile whirl-pak sample bags. Sample whirl-paks were placed on wet ice in a cooler in the field for transportation to the experimental site. The experimental site was located on a sparsely forested plot near Evaro, MT with conditions known to be suitable as elk habitat, at approximately 4000 ft elevation.

Three pellets from each animal were frozen at -20° C after arriving at the experimental site approximately 6 hours post-defecation. This initial subsample represents time-point zero samples (and technical replicates) with minimal exposure to ambient conditions typical of a direct or capture-based sampling scheme. The remaining pellets from each elk were placed in square plastic culture plates (25 cm x 25 cm) with a grid backing using sterile gloves and forceps. Each culture plate had a larger glass plate suspended above it at a height of 4 cm using a cork stopper in each corner to allow air flow and prevent direct contact with incidental precipitation (although no precipitation occurred on-site during the study), and the group of culture plates was surrounded by protective wire fencing. One plate was used for each technical replicate, with each replicate plate containing samples from all four individuals (for photos of the enclosure and a schematic of the experimental layout see **Supplementary Figure 1**). The samples were exposed to ambient conditions from March 27th through April 9th (14 days). Three samples from each elk were removed from the replicate plates after 1 day, 3 days, 7 days, and 14 days and immediately frozen at −20° C after removal from ambient conditions. A total of 60 elk pellets were experimentally collected.

Temperature was logged in 10-minute increments during the study using Thermocron temperature loggers (OnSolution Pty Ltd, Australia) distributed above and below the culture plates and shielded from direct sunlight. The temperature data were aggregated into hourly oscillations, daily max and minimum, and a smoothed average temperature. Additional temperature recordings were obtained from a NOAA weather station (Point 6, MT) 3.5 miles and 4000 ft above our site as reference.

### 2.2 Sample preparation, DNA extraction and sequencing

Frozen elk fecal pellets (stored frozen at -20° C) were prepared for DNA extractions by separating a standard weight (250 mg) cross-section from each pellet using a sterile petri dish (10 cm) and sterile safety razor blade for each sample. This fraction was placed into a designated sample tube from the Qiagen PowerSoil DNA extraction kit (Qiagen Inc., Germantown, MD) and processed using the manufacturer’s recommended protocol. The resulting purified metagenomic DNA was eluted with 100 µL PCR-grade water and stored at -20° C prior to further analysis.

To assess the bacterial community present in the fecal DNA extraction, we used a generally-conserved (i.e., “universal”) 16S/18S barcoded primer set (536F and 907R) designed to amplify the V4 and V5 variable regions of the rRNA gene (Holben et al., 2004) and PCR using 1(L of elk fecal sample metagenomic DNA standardized to 25ng/(L as template. Once amplified, samples were gel purified using the QIAGEN Gel Purification kit (QIAGEN, Germantown, MD) following the manufacturer’s recommended protocol for downstream direct sequencing. An Illumina MiSeq platform (San Diego, CA, USA) was used to obtain 300 base-pair (bp) paired-end sequencing using the Illumina MiSeq Reagent Kit.

### 2.3 Sequence and quality control

Read quality was summarized visually with FastQC and MultiQC to assess overall quality before proceeding with filtering (Andrews, 2010; Ewels et al., 2016). Primer sequences were removed using Cutadapt software and any reads without a mate-pair or recognizable primer sequence were discarded (Martin, 2011). We carried out all subsequent analyses in R (R Core Development Team, 2015) unless stated otherwise. The DADA2 package (Callahan et al., 2016) was used to quality-filter and trim paired reads according to the published workflow (Callahan, 2021). Remaining sequences were denoised and dereplicated. We next identified amplicon sequence variants (ASVs) from the resulting high-quality sequences, merged forward and reverse reads and removed chimeric sequences. ASVs were taxonomically assigned with the DADA2 instance of the Naïve-Bayes classifier and the Ribosomal Database Project II release (Cole et al., 2009). A matrix was produced containing counts corresponding to the abundance of each ASV present in each elk sample and an additional paired matrix with the taxonomic classification of each ASV.

The Phyloseq R package (McMurdie and Holmes, 2013) was used to further filter and summarize ASV tables. A small number of non-bacterial ASVs that belonged to Kingdom Archaea or in the Chloroplast Class were removed from this bacterial analysis. No mitochondrial contamination was detected. ASVs assigned to unknown phyla were removed since these are not informative for our analyses. All ASVs that failed phylogenetic assignment at the genus level were standardized to “g_unknown” in the taxonomy matrix and retained for diversity analyses.

### 2.4 Alpha diversity and hierarchical clustering

To assess the effect of time on bacterial genera and community diversity estimates, we calculated horizon plots of relative abundance (explained below), richness, alpha-diversity (Shannon and Simpson), and performed hierarchical clustering of samples. Statistical tests for richness and alpha-diversity between groups were calculated without rarefying sample data. Instead, richness at the genus and ASV level was calculated per sample with *breakaway*, which models incomplete sampling directly using flexible nonlinear regression on ratios of contiguous frequency counts and also provides standard error (SE) and p-values (Willis et al., 2020). As the *breakaway* model intercept gets close to zero (from more rare species in the frequency counts table), the estimate of the number of unobserved species increases. The *betta* function from *breakaway* was used to test for differences in sample richness (observed and unobserved diversity) across elk and time points. Alpha-diversity (Shannon and Simpson) was calculated per sample using the *DivNet* package, which incorporates diversity estimates with correction for incomplete sampling (Willis and Martin, 2021). The *betta* function was also used to calculate the significance (p-values) of the alpha-diversity estimates.

Horizon plots, from the R package *BiomeHorizon*, are useful for visualizing relative abundance change in longitudinal microbiome samples (Fink et al., 2021). To prepare this plot, the counts table was converted to relative abundance (sum scaling) and taxa were filtered to include only those present in ≥14/15 samples (i.e., 5 sampling times * 3 replicates per elk) above a threshold prevalence of 0.1% to simplify plotting. We chose to plot all genera (passing the filters) in one elk to show the effect of time across fecal microbiota and additionally we provided separate plots of *Bacteroides* and *Sporobacter* in all elk since these were the only taxa to visually change abundance (at 1% or 2% quartile abundance above or below the median abundance of the time series).

For the remaining analyses, microbial count data were transformed using the centered log-ratio (CLR) to preserve the compositional nature of the data (Gloor et al., 2017). This was done at the strain level (i.e., ASVs) and genus levels to assess the sensitivity of taxonomic level on results. All samples were hierarchically clustered to test the null hypothesis that an individual’s microbiota sampled at different time points (and replicates) are from the same probability distribution (i.e., conditions affecting the microbiota over time cannot be distinguished from time zero samples). Samples were clustered using the *hclust* function from the R stats package, using Ward’s D2 distance. The resulting trees were bootstrapped using 10,000 iterations to provide branching confidence with the *pvclust* package (Suzuki and Shimodaira, 2006). These trees were visualized and compared using the *dendextend* package (Galili, 2015).

### 2.5 Linear mixed modeling

To further examine which effects (e.g. exposure time, elk individual, and sample replication) influence the fecal microbiota, we performed a version of multivariate linear mixed modeling called LiMM-PCA (for a detailed explanation of the modeling approach see Manon and Govaerts, 2020). In brief, LiMM-PCA combines a dimension-reduction step (PCA) on the original microbial response matrix (counts) after transforming with CLR, which is then used as the response during parallel linear mixed modeling. This results in estimates of model parameters (fixed, random and residual effects), which are then interpreted by retrieving back-transformed loadings using PCA on augmented effect matrices. This also allows graphical evaluation of the importance and significance of model terms. The significance of a given effect will be measured by determining the full and null distributions of the restricted log-likelihood ratio (R)LLR test statistic by parametric bootstrap.

Since the study was structured to determine the effect of time on elk fecal microbiota, we chose to include *Day* as the first fixed effect since it is of primary interest. We measured time in a blocking design with each *Elk* (i.e., ‘patient’) designated as a random block and each time point sampled in triplicate (*Rep*). Although both *Elk* and *Rep* could justifiably be nested random effects, we chose to model *Elk* as a fixed effect since we suspected each elk might respond differently to day-of-sampling (Day : Elk; both different intercepts and slopes), and 4 biological replicates is below the recommended sample size for estimating population-level random effects. The effect of *Rep* (i.e., experiment-level replication) was modeled as a random effect since the study design contained all elk and all time points repeated three times. Treating *Rep* as an experiment-level random effect provided 20 data points per experiment. The final model was ~Day+*Elk*+*Day*:*Elk*+(1|*Rep*). P-values were obtained by 2,000 parametric bootstrap iterations of the (R)LLR test statistic for each model term.

## 3 Results

### 3.1 Environmental variables

Temperature was logged in 10-minute increments over the 14-day study using Thermochron temperature loggers (Baulkham Hills, AU). The temperature data were aggregated into hourly oscillations, daily maximum and minimum, and a smoothed average temperature (**Supplementary Figure 2**). Additional temperature recordings from a NOAA weather station Point 6, 3.5 miles and 4000 ft above our site recorded expectedly lower temperatures but an identical trend to the localized temperature loggers (**Supplementary Data** not shown). Precipitation did not occur in measurable amounts during the study.

### 3.2 Sequencing and quality control

Sequencing and quality control of partial 16S rRNA amplicons from each fecal sample provided a total of 5,210,542 paired-end sequences (forward and reverse reads) across all elk sampled, with sample depths varying from 23,800 to 267,983 sequences per individual (mean depth 86,842). Primers were trimmed from paired-end sequences with filtering parameters that required a read to have a primer present, a minimum length of 100 base-pairs (bp), and a mate-pair, which resulted in 5,172,731 sequences remaining. Filtering and trimming on quality (max of 2 errors, no unidentified bases [i.e. ‘Ns’] and sequences truncated at the instance of quality = 2) and length (minimum of 80 bp after quality trimming) reduced the number of sequences retained to 3,793,295. The remaining sequences were dereplicated and amplicon sequence variants (ASVs) were inferred independently with forward and reverse reads using the DADA2 error model. Forward and reverse reads were merged into single reads (2,384,561 remaining), Chimeric sequences were removed, resulting in 2,158,150 high-quality paired sequences remaining. Final read depths per elk ranged from 7,812 to 126,887 (mean = 35,969 with just two samples below 10,000 reads) after these quality filters. Sequence filtering results are summarized across samples and per sample in **Supplementary Table 1**.

### 3.3 Microbiota diversity and characterization

Sequencing of partial 16S rRNA gene amplicons from each fecal sample provided a survey of bacterial presence and abundance. ASV counts per animal ranged from 7,799 to 126,043 after quality filtering, with a mean count of 35,772. After filtering, a total of 5,177 unique ASVs were indicated across all samples. The distribution of unique ASVs (observed richness) within individual samples ranged from 301 to 1965, with a mean of 850 unique ASVs per individual (see **Supplementary Table 1**). After assigning phylogeny to ASVs, there were 112 unique identified genera and 7 identified species (we note that 16S gene phylogeny has limited resolution to the species level). The top 10 most abundant ASVs across all samples were classified to the genera *Sporobacter, Prevotella, Bacteroides*, or were “g(enus):Unknown”, all within the phyla Bacteroidota or Bacillota.

**Figure 1:**
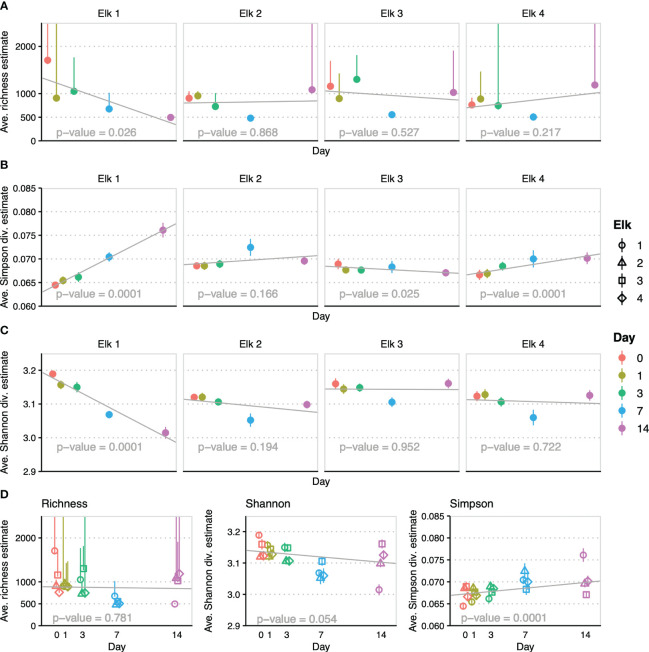
Modeled breakaway alpha diversity and richness estimates colored by day of sampling: Shapes represent each individual elk and colors represent day of sampling where appropriate. **(A)** Plots of breakaway richness estimates (points) and SD of triplicate samples (whiskers) by day of sampling with trend line and trend significance (reported inset on x-axis throughout) per elk. **(B)** Plots of breakaway Simpson diversity estimates (points) and SD of triplicate samples (whiskers) by day of sampling with trend line and trend significance per elk. **(C)** Plots of breakaway Shannon diversity estimates (points) and SD of triplicate samples (whiskers) by day of sampling with trend line and trend significance per elk. **(D)** Plots combining all elk samples by day of sampling for demonstration of overall trend and significance for Richness, Shannon and Simpson models.

Richness estimates modeled in *breakaway* are defined as the total diversity including the unobserved (unsampled) count (Willis et al., 2020). Richness estimates were calculated at the genus level per elk and time point and are summarized in **Figure 1A**. Richness variation within individual elk across time replicates was not significant (p > 0.2). This lack of significance is even more pronounced (p = 0.781) when all elk were modeled together (**Figure 1D**). The *breakaway* Shannon and Simpson diversity estimates (**Figures 1B, C**) were calculated similarly for each elk and time point and then combined in a global model for time (**Figure 1D**). Simpson diversity is more sensitive to rare taxa and was significant in 3 elk, although one significant trend had a sign change (negative slope, higher diversity) compared to the other significant trends (positive slopes, lower diversity) (**Figure 1B**). The slight positive trend for Simpson diversity (a decrease in diversity, sensitive to rare taxa) across sampling days was significant in the global model (p = 0.0001). Shannon diversity gives more weight to evenness and common species, which was apparent with a lack of significance in all but “elk-1”, as well as a slightly negative trend (p = 0.054) in the global model, which was heavily influenced by elk-1 (**Figure 1C**).

**Figure 2:**
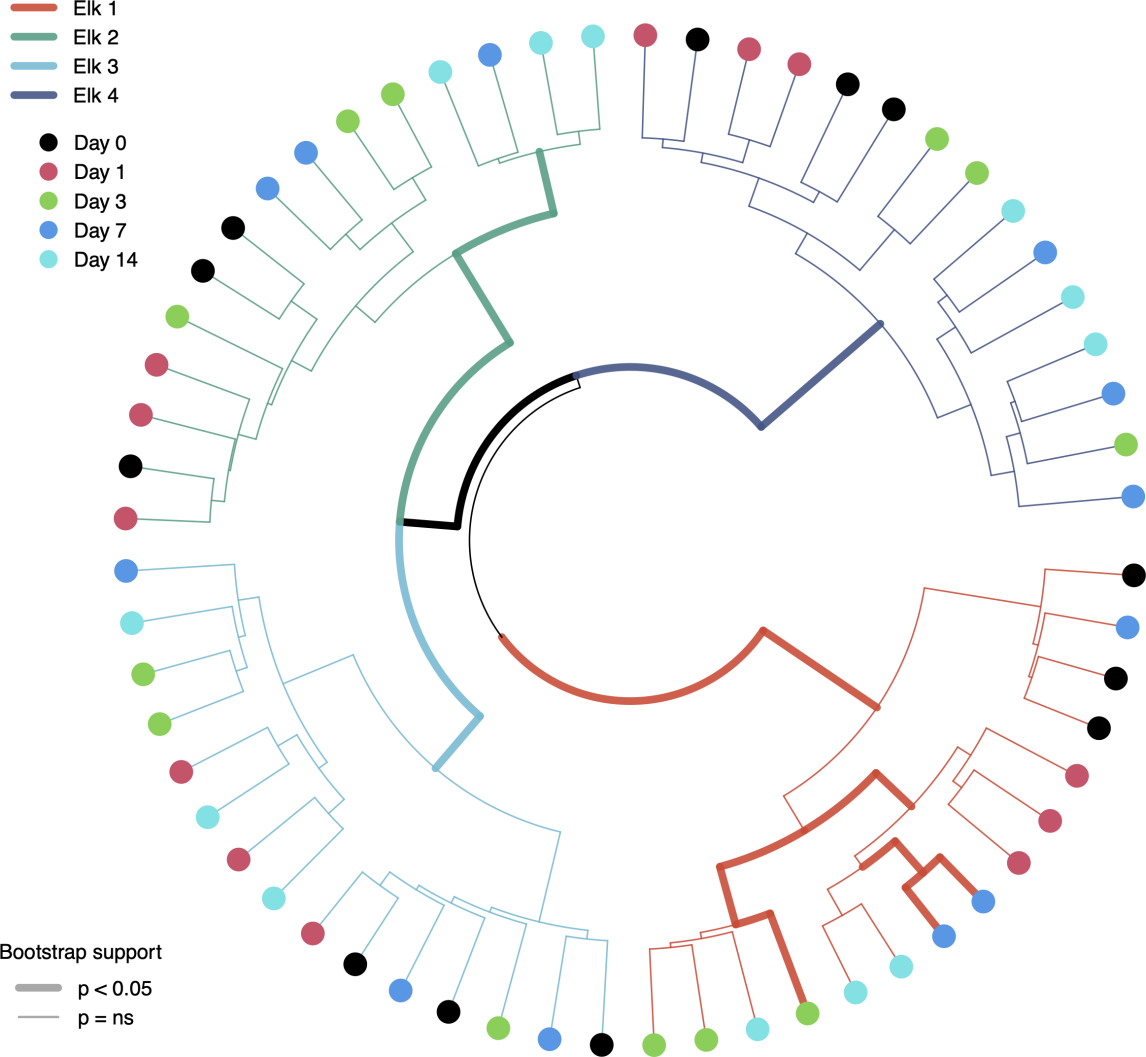
Unsupervised clustering of elk fecal microbiota samples using Ward’s D2 method. Line colors represent samples of each elk, and line weight represents significance level of branching using 10,000 bootstrap iterations. Node colors depict the day samples were collected. Note the lack of consistent clustering by day of sampling but the complete clustering and significance of sample source (individual elk).

To visualize changes in relative abundance of bacterial genera across time, we produced horizon plots as an alternative to stacked bar plots since they better visualize small changes in a series (**Figure 2**). These results show little change (+/- generally on the order of 1%) across common genera with the exception of *Sporobacter* and *Bacteroides* which show a subtle increase and decrease across time, respectively. These genus-specific changes in abundance are based on the median abundance calculated across the time series using 2% quartiles, and we suggest that a change of 2-4% over 7 days in two genera is not inordinate in the context of the entire community. The abundance differences seen at day 14 (+/- 10%), while more substantial, are still proportionally small overall.

### 3.4 Hierarchical clustering

**Figure 3:**
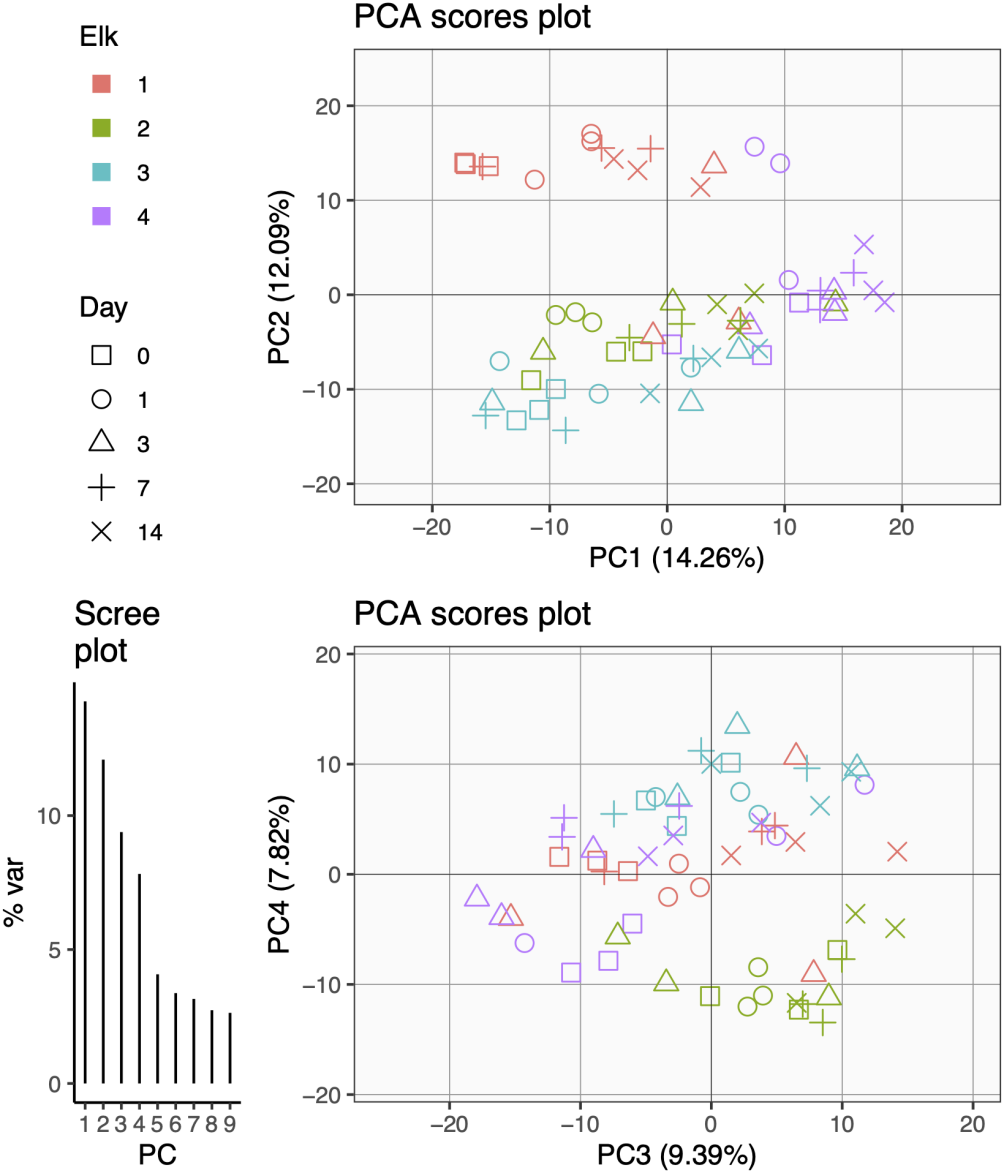
Beta diversity: Aitchison’s distance (Euclidean) PCA plots of elk fecal microbiota samples transformed with centered log-ratio (CLR) and corresponding axis loadings. Only principal components (PCs) with variation explained above 5% are included. Top: PCA scores plot of PC1 and PC2 axes. Bottom: PCA Scores plot of PC3 and PC4 axes. Shapes depict day of sampling colored by elk. Lower left: Scree plot of variance per PC.

To test the null hypothesis that individual time points and replicates come from the same probability distribution (i.e., conditions affecting the elk fecal microbiota over time cannot be distinguished), we hierarchically clustered centered log-ratio (CLR)-transformed samples with 10,000 bootstrap iterations. The result of unsupervised clustering showed significant (p < 0.05) and complete separation of individual elk, with all respective elk sub-samples falling within the same branch (**Figure 3**). Interestingly, there was virtually no support for clustering of leaves or nodes (i.e. day-of-sampling and individual sample replicates) on each elk branch at an alpha of either 0.05 or 0.1 (**Figure 3**). Elk-1 showed the best clustering by sample day (and the only significant leaf results), corroborating the more extreme changes seen in its richness and alpha-diversity estimates, but the plotted representation is only one of 10,000 iterations and should not be interpreted as representative of leaf clustering since overall, it was not significant.

### 3.5 LiMM-PCA models

**Figure 4:**
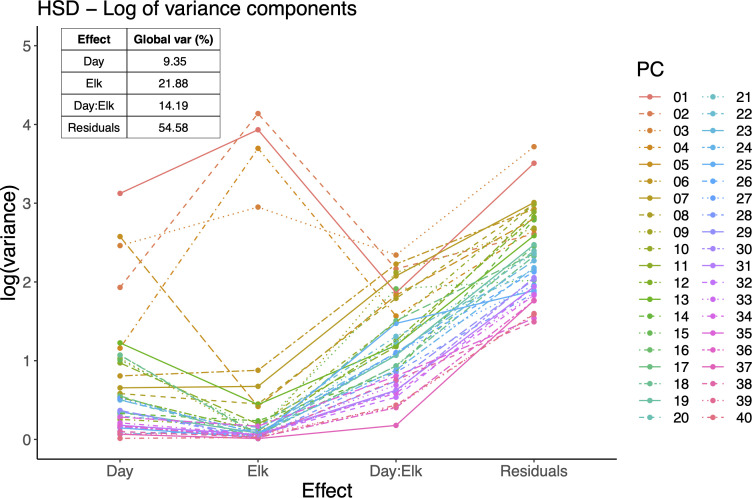
Variance components of LiMM-PCA mixed model. Main plot shows the log variance explained by each model term for each PC, including the interaction term and residuals. Inset right: sum of global variance explained by model terms and residuals. All PCs are included thus variance percentages sum to 100.

To further identify which effects (i.e., time, elk, individual sample replication) influence the fecal microbiota in elk sampled non-invasively across time, we performed LiMM-PCA (Manon and Govaerts, 2020). This analysis produced PCA plots of fecal microbiota samples transformed with CLR and their corresponding axis loadings. The first PCA on the CLR microbial abundance matrix (prior to modeling) captured <45% of variance in the first 4 PCs (**Figure 4**). This relatively low percentage of variance explained speaks to the typical high noise found in microbiome datasets. Similar to hierarchical clustering results, the PCA clustering of samples using either {PC1, PC2} or {PC3, PC4} was strongly descriptive of elk replicates (colors) and showed relatively low importance of sampling day (shapes) (**Figure 4**).

**Figure 5:**
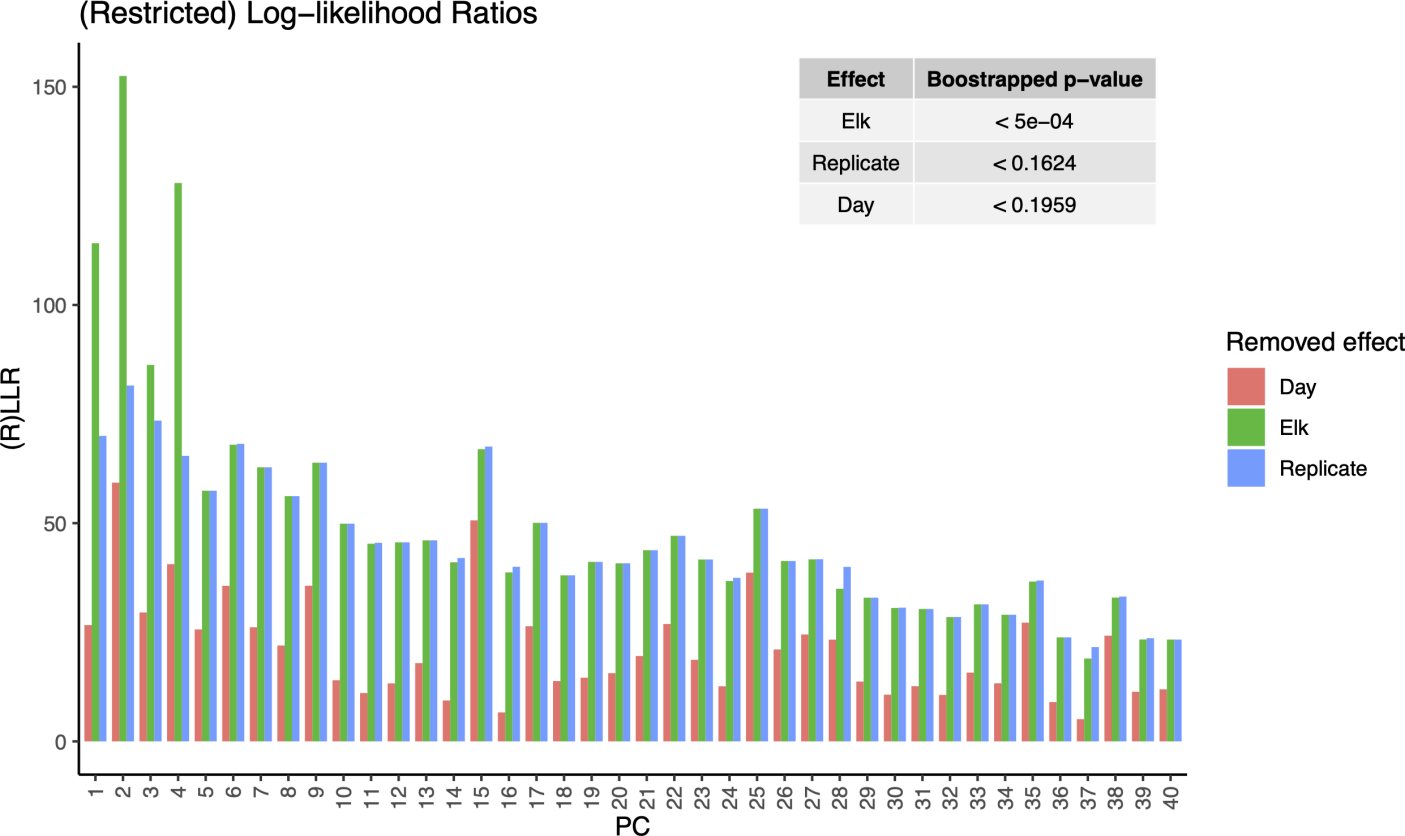
Restricted Log-likelihood ratios (RLLR) of LiMM-PCA mixed models. Main plot shows the restricted log likelihood of day-of-sampling (Day), Elk, and Replicate. Inset table: Bootstrapped p-values of model terms using 2000 iterations. Elk was the only significant model variable and most of the model variance was contained in the first 4 PCs.

**Figure 6:**
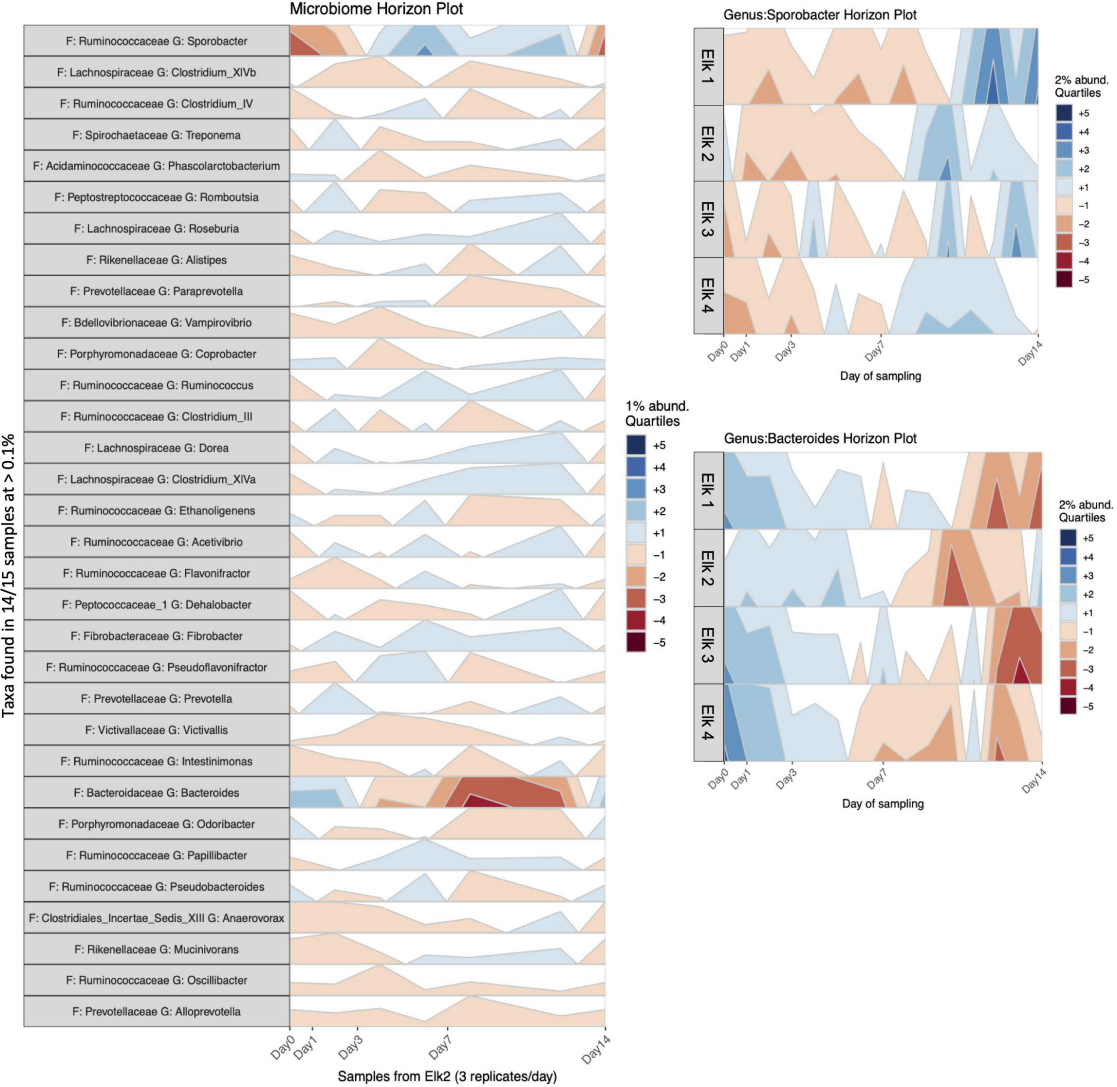
Horizon plots of longitudinal elk fecal microbiota samples. Left: Horizon plot of longitudinal elk2 microbiota samples. Taxa were filtered to include only those present in 14/15 samples (5 samples * 3 reps) above a threshold prevalence of 0.1%. The y-axis is the relative abundance of a taxon (genera) at a given time point. Colors indicate the 1% quartile abundance above (blue) or below (red) the median abundance of the time series. Right: Bacteroides and Sporobacter genera in each elk (triplicate sample abundances were averaged). Colors indicate the 2% quartile abundance above (blue) or below (red) the median abundance of the time series.

The LiMM-PCA model variance components (i.e., PCs) show the respective contribution of variance for each model term (**Figure 5**). We saw that, like the initial PCA, the total variance explained by PCs 1-4 contribute the most to the model, with most other PCs contributing to the residual error. Of the model terms, *Elk* had the largest influence with 21.88% of global variance, *Day : Elk* interaction with 14.9% and *Day* with 9.35%. The fixed terms (*Day, Elk*) and random term (*Rep*) were tested for significance using 2000 bootstrap iterations of (R)LLR statistic and only *Elk* was found to be significant (**Figure 6**).

A visual inspection of the modeling results and loadings using PCA on each effect matrix is provided in supplemental materials and shows the pattern described by each model term and the subset of ASVs impacting this pattern (**Supplementary Figure 3**). The ASV’s with high loading scores could be interrogated per model term to provide additional information and future hypotheses, but this was outside the scope of our study. The PCA scores on the raw CLR-transformed data (**Figure 3**) show strong separation by elk and, unsurprisingly, this is repeated in the augmented matrix (**Supplementary Figure 3**). Perhaps what is surprising is the clustering of day-of-sampling which does show separation based on time of sampling, although not significant. The effect of replication on the samples accounts for only a small amount of variation.

## 4 Discussion

The reliable use of non-invasive fecal sampling for wildlife monitoring hinges on the stability of the fecal microbiota community across time under ambient conditions. In this longitudinal experiment, we showed that the relative abundance of taxa is not significantly biased by varying field conditions and age of sample for at least 7 days. In light of these findings, the distinctive value of non-invasive fecal microbiota samples in augmenting current wildlife monitoring techniques is further verified.

In this experiment we specifically asked: Are fecal microbiota communities in elk stable across intervals typically used for non-invasive sampling? Although we conclude that elk fecal microbiota are stable within 1 week, over the 7-14 day interval there are some caveats. The phylogenetic level of amplicon variation (e.g., genus or ASV) being used is important in interpreting the response of the microbiome to sample age. Higher phylogenetic levels like genus examined herein, seem relatively unperturbed by time, especially within one week of defecation. Yet, we observed strain level diversity (derived from ASVs) to be somewhat noisy, even among technical replicates (**Supplementary Figure 4**). We suggest that ASVs may suffer from additional sources of variation that plague microbiome studies in general, such as sources of technical variation including DNA extraction bottlenecks (e.g., from the tendency to saturate extraction columns with abundant DNA preventing low abundance representation) and sequence depth. Further, ASV’s may be sensitive to potential sources of biological variation such as DNA degradation and micro-site effects. Although strain level diversity was not tested here (and is usually not required or recommended for wildlife microbiome characterization), these sources of variation should be considered if strain level diversity is important for a prospective study.

Another question asked was: Does sample age influence the results of commonly measured diversity estimates and sample clustering analyses? We showed that diversity estimates are relatively stable but may decline slightly over time. However, the data suggest that the magnitude of decline is not biologically significant since sign changes in the slopes of the relationship were seen to differ between biological replicates, suggesting a lack of a mechanistic or concerted response. A more likely explanation is stochastic differences in the original community composition of the fecal pellets themselves (i.e., not all pellets from the same animal sampled from the droppings pile are strictly identical to begin with). We also found that diversity estimates are sensitive to outlier individuals and samples (e.g., elk-1). This outlier effect is problematic for any study, but especially for non-invasive studies with modest biological replication. We suggest outlier samples can be overcome in the traditional way by simply examining larger sample sizes and removing outliers based on statistical criteria if necessary. Our clustering analysis clearly showed that variation is largely contained at the individual level and is not significant among subsamples, but outlier effects are also pervasive in these analyses, although not significant.

The last question addressed in this study sheds light on the variance attributed to each variable in our structured sample design, namely: What proportion of the variance in fecal microbiota composition is attributed to day-of-sampling, individuals, and replicates? The ability to graphically illustrate the effects of each variable and their significance using LiMM-PCA proved to be a valuable tool for further evaluating the nuances of sample age on the fecal microbiota and the taxa driving these relationships. The relatively small and non-significant variance attributed to the effect of *Day* supports the conclusion that non-invasive sampling is robust to sample age. The significant variance attributed to each elk and the additional variance captured by the interaction of *Elk : Day* strongly supports that fecal microbiota composition consistently and primarily reflect the animal’s unique signature of variation, despite sample age. That said, this may be a strength to this general, non-invasive approach where differing ecological parameters between individual animals such as age, body-fat composition, population/herd, or sex are of importance (Pannoni et al., 2022). Although a large proportion of variance is described by the interaction term, it was not significant, suggesting that the changes due to sample age are largely unique to each biological replicate and are not indicative of a mechanistic response to fecal pellet age, such as what might be seen from rapid overgrowth or turnover in one or few genera in all samples. Pseudo-replication (i.e., *Rep*) of each individual was found to be non-significant and suggests that between-pellet variation from a single individual (i.e., technical variation) is minimal in this study. In our earliest preliminary studies, we assessed whether subsamples of individual pellets (i.e. single pellet fractions) exhibited community structure variance. There, we found that intra-pellet variation had undetectable differences as determined by DGGE analysis of partial rRNA gene amplicons from purified metagenomic DNA (unpublished observations).

Although specific to elk and likely other ungulates, we expect this experiment will be illuminating to investigators of various wildlife microbiome systems in temperate northern latitudes where winter/spring conditions are similar to our experimental setting. The results should not be extrapolated to other climates, environments or non-ungulate study species without experimental verification. Of more general relevance is the process and methods of identifying potential microbiome biases due to the specifics of non-invasive sample acquisition, which should be common practice. We also highlight LiMM-PCA as a modeling technique that is of particular value for microbiome studies, which often have hierarchical sampling designs, repeat measures and a multivariate response (Manon and Govaerts, 2020). LiMM-PCA controls for random effects (e.g., the patient effect) and structured study designs (repeat measures) using linear mixed models and allows for dimension reduction of the microbial response matrix by PCA. In this regard, LiMM-PCA is superior to other ANOVA approaches which do not have this flexibility especially with unbalanced data, and we encourage its broader use specifically for longitudinal microbiome studies with structured study designs. We used an alternative method, *breakaway*, to estimate richness and diversity that does not require rarefaction of samples to a common sampling depth which may be of general interest to the field. Breakaway (and DivNet) use statistical (non-linear) models fit to each sample’s unique distribution of species counts to determine how much larger estimates of richness should be (Willis et al., 2020; Willis and Martin, 2021).

This study is not intended as an authoritative example of best practices when modeling longitudinal microbiomes considering the potential for unique challenges of individual study designs. However, we believe that our experiment can help encourage the broader use of linear mixed models for multivariate outcomes in microbiome research by highlighting its power to visualize variance relationships and obtain significance for variables of interest. Quantifying and isolating sources of variation in elk fecal microbiota due to sample age and the taxa driving these changes will allow more thoughtful interpretation of various diversity estimates and differentially abundant taxa in other related studies where time to sampling cannot be strictly controlled.

## Data availability statement

The original datasets and contributions presented in this study are publicly available. The raw data can be found at EMBL-EBI (www.ebi.ac.uk) under accession ERP157099. All sequence files are also on DRYAD here: https://doi.org/10.5061/dryad.v6wwpzh2b. The code used for all analyses is publicly available at the following link: https://github.com/samasafish/elk_microbiota_time_series_Frontiers.git.

## Ethics statement

Ethical approval was not required for the study involving animals in accordance with the local legislation and institutional requirements because samples were collected non-invasively without animal perturbation.

## Author contributions

SP: Conceptualization, Data curation, Formal analysis, Investigation, Methodology, Validation, Visualization, Writing – original draft, Writing – review & editing, Funding acquisition. WH: Conceptualization, Investigation, Methodology, Project administration, Supervision, Writing – original draft, Writing – review & editing, Funding acquisition, Resources.

## References

[B1] Amato K. R. Sanders G. Song S. J. Nute M. Metcalf J. L. Thompson L. R. . (2019). Evolutionary trends in host physiology outweigh dietary niche in structuring primate gut microbiomes. ISME J. 13 (3), 576–587. doi: 10.1038/s41396-018-0175-0 29995839 PMC6461848

[B2] Andrews S. (2010). FastQC: A quality control tool for high throughput sequence data.

[B3] Arrieta J. M. Weinbauer M. G. Herndl G. J. (2000). Interspecific variability in sensitivity to UV radiation and subsequent recovery in selected isolates of marine bacteria. Appl. Environ. Microbiol. 66 (4), 1468–1473. doi: 10.1128/AEM.66.4.1468-1473.2000/ASSET/88723381-ADB0-4570-91DD-07A16B22A6B4/ASSETS/GRAPHIC/AM0401803005.JPEG 10742228 PMC92009

[B4] Brusa T. Canzi E. Pacini N. Zanchi R. Ferrari A. (1989). Oxygen tolerance of anaerobic bacteria isolated from human feces. Curr. Microbiol. 19 (1), 39–43. doi: 10.1007/BF01568901

[B5] Callahan B. J. (2021). DADA2 Pipeline Tutorial (1.16) (GitHub), 1. Available at: https://benjjneb.github.io/dada2/tutorial.html.

[B6] Callahan B. J. McMurdie P. J. Rosen M. J. Han A. W. Johnson A. J. A. Holmes S. P. (2016). DADA2: High-resolution sample inference from Illumina amplicon data. Nat. Methods 13 (7), 581–583. doi: 10.1038/nmeth.3869 27214047 PMC4927377

[B7] Cardona S. Eck A. Cassellas M. Gallart M. Alastrue C. Dore J. . (2012). Storage conditions of intestinal microbiota matter in metagenomic analysis. BMC Microbiol. 12, 158. doi: 10.1186/1471-2180-12-158 22846661 PMC3489833

[B8] Carroll I. M. Ringel-Kulka T. Siddle J. P. Klaenhammer T. R. Ringel Y. (2012). Characterization of the fecal microbiota using high-throughput sequencing reveals a stable microbial community during storage. PloS One 7 (10), e46953. doi: 10.1371/journal.pone.0046953 23071673 PMC3465312

[B9] Cole J. R. Wang Q. Cardenas E. Fish J. Chai B. Farris R. J. (2009). The Ribosomal Database Project: improved alignments and new tools for rRNA analysis. Nucleic Acids Res. 37 (suppl_1), D141–D145. doi: 10.1093/nar/gkn879 19004872 PMC2686447

[B10] Corl A. Charter M. Rozman G. Toledo S. Turjeman S. Kamath P. L. . (2020). Movement ecology and sex are linked to barn owl microbial community composition. Mol. Ecol. 29 (7), 1358–1371. doi: 10.1111/mec.15398 32115796

[B11] Ewels P. Magnusson M. Lundin S. Käller M. (2016). MultiQC: Summarize analysis results for multiple tools and samples in a single report. Bioinformatics 32 (19), 3047–3048. doi: 10.1093/bioinformatics/btw354 27312411 PMC5039924

[B12] Fink I. Abdill R. J. Blekhman R. Grieneisen L. (2021). BiomeHorizon: visualizing microbiome time series data in R. MSystems 7 (3). doi: 10.1101/2021.08.29.458140 PMC9238406 35499306

[B13] Galili T. (2015). dendextend: an R package for visualizing, adjusting and comparing trees of hierarchical clustering. Bioinf. (Oxford England) 31 (22), 3718–3720. doi: 10.1093/BIOINFORMATICS/BTV428 PMC4817050 26209431

[B14] Gaulke C. A. Arnold H. K. Humphreys I. R. Kembel S. W. O’dwyer J. P. Sharpton T. J. (2018). Ecophylogenetics clarifies the evolutionary association between mammals and their gut microbiota. MBio 9 (5). doi: 10.1128/mBio.01348-18 PMC6134092 30206171

[B15] Gelman A. (2005). Analysis of variance-why it is more important than ever. Ann. Stat 33 (1), 1–31. doi: 10.2307/3448650

[B16] Gloor G. B. Macklaim J. M. Pawlowsky-Glahn V. Egozcue J. J. (2017). Microbiome datasets are compositional: and this is not optional. Front. Microbiol. 8. doi: 10.3389/fmicb.2017.02224 PMC5695134 29187837

[B17] Hale V. L. Tan C. L. Niu K. Yang Y. Cui D. Zhao H. . (2016). Effects of field conditions on fecal microbiota. J. Microbiol. Methods 130, 180–188. doi: 10.1016/j.mimet.2016.09.017 27686380

[B18] Holben W. E. Feris K. P. Kettunen A. Apajalahti J. H. (2004). GC fractionation enhances microbial community diversity assessment and detection of minority populations of bacteria by denaturing gradient gel electrophoresis. J. Appl. Environ. Microbiol. 70 (4), 2263–2270. doi: 10.1128/AEM.70.4.2263-2270.2004 PMC383056 15066821

[B19] Huus K. E. Ley R. E. (2021). Blowing hot and cold: body temperature and the microbiome. MSystems 6 (5). doi: 10.1128/MSYSTEMS.00707-21 PMC8552956 34581596

[B20] Ingala M. R. Simmons N. B. Wultsch C. Krampis K. Speer K. A. Perkins S. L. (2018). Comparing microbiome sampling methods in a wild mammal: Fecal and intestinal samples record different signals of host ecology, evolution. Front. Microbiol. 9 (MAY). doi: 10.3389/fmicb.2018.00803 PMC5938605 29765359

[B21] Kim D. Hofstaedter C. E. Zhao C. Mattei L. Tanes C. Clarke E. . (2017). Optimizing methods and dodging pitfalls in microbiome research. Microbiome 5 (1). doi: 10.1186/s40168-017-0267-5 PMC5420141 28476139

[B22] Knutie S. A. Chaves J. A. Gotanda K. M. (2019). Human activity can influence the gut microbiota of Darwin’s finches in the Galapagos Islands. Mol. Ecol. 28 (9), 2441–2450. doi: 10.1111/mec.15088 31021499

[B23] Lauber C. L. Zhou N. Gordon J. I. Knight R. Fierer N. (2010). Effect of storage conditions on the assessment of bacterial community structure in soil and human-associated samples. FEMS Microbiol. Lett. 307 (1), 80–86. doi: 10.1111/j.1574-6968.2010.01965.x 20412303 PMC3148093

[B24] Lynch J. B. Hsiao E. Y. (2019). Microbiomes as sources of emergent host phenotypes. Science 365 (6460), 1405–1409). doi: 10.1126/science.aay0240 31604267

[B25] Manon M. Govaerts B. (2020). LiMM-PCA: Combining ASCA+ and linear mixed models to analyse high-dimensional designed data. J. Chemom. 34 (6), e3232. doi: 10.1002/cem.3232

[B26] Martin M. (2011). Cutadapt removes adapter sequences from high-throughput sequencing reads. EMBnet. Journal 17 (1), 10. doi: 10.14806/ej.17.1.200

[B27] Mata V. A. Rebelo H. Amorim F. McCracken G. F. Jarman S. Beja P. (2019). How much is enough? Effects of technical and biological replication on metabarcoding dietary analysis. Mol. Ecol. 28 (2), 165–175. doi: 10.1111/mec.14779 29940083 PMC7379978

[B28] McCambridge J. McMeekin T. A. (1981). Effect of solar radiation and predacious microorganisms on survival of fecal and other bacteria. Appl. Environ. Microbiol. 41 (5), 1083–1087. doi: 10.1128/AEM.41.5.1083-1087.1981 7020590 PMC243871

[B29] McMurdie P. J. Holmes S. (2013). phyloseq: an R package for reproducible interactive analysis and graphics of microbiome census data. PloS One 8 (4), e61217. doi: 10.1371/journal.pone.0061217 23630581 PMC3632530

[B30] Menke S. Meier M. Sommer S. (2015). Shifts in the gut microbiome observed in wildlife faecal samples exposed to natural weather conditions: Lessons from time-series analyses using next-generation sequencing for application in field studies. Methods Ecol. Evol. 6 (9), 1080–1087. doi: 10.1111/2041-210X.12394

[B31] Pannoni S. B. Proffitt K. M. Holben W. E. (2022). Non-invasive monitoring of multiple wildlife health factors by fecal microbiome analysis. Ecol. Evol. 12 (2), e8564. doi: 10.1002/ece3.8564 35154651 PMC8826075

[B32] R Core Development Team . (2015). “R: a language and environment for statistical computing, 3.2.1,” in Document freely available on the internet at: http://www.r-project. org (R Foundation for Statistical Computing). doi: 10.1017/CBO9781107415324.004

[B33] Roesch L. F. W. Casella G. Simell O. Krischer J. Wasserfall C. H. Schatz D. . (2009). Influence of fecal sample storage on bacterial community diversity. Open Microbiol. J. 3 (1), 40–46. doi: 10.2174/1874285800903010040 19440250 PMC2681173

[B34] Suzuki R. Shimodaira H. (2006). Pvclust: an R package for assessing the uncertainty in hierarchical clustering. Bioinf. (Oxford England) 22 (12), 1540–1542. doi: 10.1093/BIOINFORMATICS/BTL117 16595560

[B35] Taschuk R. Griebel P. J. (2012). Commensal microbiome effects on mucosal immune system development in the ruminant gastrointestinal tract. *Anim. Health Res. Rev*. 13 (1), 129–141. doi: 10.1017/S1466252312000096 22853940

[B36] Tzeneva V. A. Salles J. F. Naumova N. de Vos W. M. Kuikman P. J. Dolfing J. . (2009). Effect of soil sample preservation, compared to the effect of other environmental variables, on bacterial and eukaryotic diversity. Res. Microbiol. 160 (2), 89–98. doi: 10.1016/j.resmic.2008.12.001 19111612

[B37] Wang L. Zhang G. Li Y. Zhang Y. (2020). Effects of high forage/concentrate diet on volatile fatty acid production and the microorganisms involved in VFA production in cow rumen. Animals 10 (2), 223. doi: 10.3390/ANI10020223 32019152 PMC7070707

[B38] Willis A. Martin B. D. (2021). DivNet: Diversity Estimation in Networked Ecological Communities. (R package version 0.3.7.).

[B39] Willis A. Martin B. D. Trinh P. Teichman S. Barger K. Bunge J. (2020). breakaway: Species Richness Estimation and Modeling. (R package version 4.7.3).

[B40] Wu G. D. Chen J. Hoffmann C. Bittinger K. Chen Y.-Y. Keilbaugh S. A. . (2011). Linking long-term dietary patterns with gut microbial enterotypes. Sci. (New York N.Y.) 334 (6052), 105–108. doi: 10.1126/science.1208344 PMC3368382 21885731

[B41] Zhu L. Wang J. Bahrndorff S. (2021). Editorial: the wildlife gut microbiome and its implication for conservation biology. Front. Microbiol. 12. doi: 10.3389/fmicb.2021.697499 PMC8256134 34234768

