# Supplementary material for: Wildlife fecal microbiota exhibit community stability across a longitudinal semi-controlled non-invasive sampling experiment
Source: Front Microbiomes. 2024 Feb 13;3:1274277. doi: 10.3389/frmbi.2024.1274277 (PMC12993598; doi:10.3389/frmbi.2024.1274277)
Supplement: Supplementary file 1 [file DataSheet_1.docx]

Supplementary Material

# Supplementary Figures and Tables

## Supplementary Figures

**

**Supplementary Figure 1.** Visual experimental design and sampling plots. A) Shows 3 gridded culture plates where elk fecal pellets were aged in triplicate; B) A picture of final ageing conditions with a glass plate protecting pellets from incidental precipitation and the placement of Thermocron temperature sensors (silver buttons visible between glass plates, not all sensors are all visible). C) Visual representation of experimental design of longitudinal elk replicates across plates and time of sampling.

**Supplementary Figure 2.** Recorded temperature during 14-day study. Lines: daily oscillating temperature and smoothed average indicated by colored solid-lines and typical rumen temperature indicated by dashed red-line for reference. NOAA weather station data (not shown) was used for trend verification only because differences between local temperatures and NOAA data was expected due to elevational differences (~2000 ft).

**Supplementary Figure 3.** Individual PCA on the effect matrices for each model variable and the residual effects. From left to right: scree plots, scores, and loadings of {PC1, PC2}. The scree plots and loadings plots are from PCA on the pure effect matrices whereas the scores are augmented.

**Supplementary Figure 4.** A comparison of ASV and Genus level taxa shared among sample days for each elk. Plots show that taxonomic level is important for describing a response to sample age depending on the goal of the study. ASVs are noisy and may be driven by other sources of variation. Genus level diversity remains stable through time.

## Supplementary Tables

**Supplementary Table 1.** Sample filtering totals for each quality control step.

**

**Supplementary Table 2.** Sequence read summary: This table describes the processing of raw reads based on quality prior to assessing microbial diversity among samples.

|  | Min | Median | Mean | Max | Total |
| --- | --- | --- | --- | --- | --- |
| Raw read pairs | 23,800 | 65,786 | 86,842 | 267,983 | 5,210,542 |
| Primer trim & filter | 23,590 | 65,264 | 86,212 | 265,897 | 5,172,731 |
| Quality filter & trim | 16,970 | 47,724 | 63,222 | 196,979 | 3,793,295 |
| Denoise Fwd. | 15,563 | 45,664 | 60,626 | 192,614 | 3,637,573 |
| Denoise Rev. | 16,069 | 46,296 | 61,441 | 194,089 | 3,686,477 |
| Merged Fwd. & Rev. | 8,383 | 30,424 | 39,743 | 142,648 | 2,384,561 |
| Non-chimeric reads | 7,812 | 28,062 | 35,969 | 126,887 | 2,158,150 |
